# The Influence of *OLR1* and *PCSK9* Gene Polymorphisms on Ischemic Stroke: Evidence from a Meta-Analysis

**DOI:** 10.1038/srep18224

**Published:** 2015-12-15

**Authors:** Anthony Au, Lyn R. Griffiths, Kian-Kai Cheng, Cheah Wee Kooi, Looi Irene, Loo Keat Wei

**Affiliations:** 1 Institute of Bioproduct Development and Department of Bioprocess Engineering, Faculty of Chemical Engineering, Universiti Teknologi Malaysia, 81300 Johor, Malaysia; 2 Genomics Research Centre, Institute of Health and Biomedical Innovation, Queensland University of Technology, Musk Avenue, Kelvin Grove, QLD 4059, Australia; 3 Innovation Centre in Agritechnology, Universiti Teknologi Malaysia, 81300 Johor, Malaysia; 4 Department of Medicine, Taiping Hospital, Jalan Tamingsari, 34000 Taiping, Perak, Malaysia; 5 Medical Department and Clinical Research Centre, Hospital Seberang Jaya, Jalan Tun Hussein Onn, 13700 Seberang Jaya, Pulau Pinang, Malaysia; 6 Centre for Biodiversity Research, Universiti Tunku Abdul Rahman, Bandar Barat, 31900 Kampar, Perak, Malaysia; 7 Department of Biological Science, Faculty of Science, Universiti Tunku Abdul Rahman, Bandar Barat, 31900 Kampar, Perak, Malaysia

## Abstract

Both *OLR1* and *PCSK9* genes are associated with atherosclerosis, cardiovascular disease and ischemic stroke. The overall prevalence of *PCSK9* rs505151 and *OLR1* rs11053646 variants in ischemic stroke were 0.005 and 0.116, respectively. However, to date, association between these polymorphisms and ischemic stroke remains inconclusive. Therefore, this first meta-analysis was carried out to clarify the presumed influence of these polymorphisms on ischemic stroke. All eligible case-control and cohort studies that met the search terms were retrieved in multiple databases. Demographic and genotyping data were extracted from each study, and the meta-analysis was performed using RevMan 5.3 and Metafor R 3.2.1. The pooled odd ratios (ORs) and 95% confidence intervals (CIs) were calculated using both fixed- and random-effect models. Seven case-control studies encompassing 1897 cases and 2119 controls were critically evaluated. Pooled results from the genetic models indicated that *OLR1* rs11053646 dominant (OR = 1.33, 95%  CI:1.11–1.58) and co-dominant models (OR = 1.24, 95%  CI:1.02–1.51) were significantly associated with ischemic stroke. For the *PCSK9* rs505151 polymorphism, the OR of co-dominant model (OR = 1.36, 95%  CI:1.01–1.58) was found to be higher among ischemic stroke patients. In conclusion, the current meta-analysis highlighted that variant allele of *OLR1* rs11053646 G > C and *PCSK9* rs505151 A > G may contribute to the susceptibility risk of ischemic stroke.

Ischemic stroke is a heterogeneous group of neurovascular diseases and contributes to major morbidity and mortality in both developed and developing countries^1^. The major risk factors for ischemic stroke such as obesity, diabetes mellitus, hypertension, hypercholesterolemia, dyslipidemia and atherosclerosis are well established^2^. Nevertheless, the mechanism of ischemic stroke has not been fully elucidated and may involve a complex interplay between environmental and genetic factors, such as polymorphic variants of the genes that regulate cholesterol and lipid biosynthesis or degradation^2^. Emerging lines of evidence revealed that lectin-like oxidized-low density lipoprotein receptor-1 (LOX-1) and proprotein convertase subtilisin/kexin 9 (PCSK9) play critical roles in hyperlipidemia and atherogenesis development, that ultimately leads to ischemic stroke^3^,^4^.

LOX-1 is one of the major scavenger receptor for oxidized low density lipoprotein (ox-LDL), which encoded by human oxidized low density lipoprotein receptor 1 (*OLR1*) gene. This receptor protein mediates the recognition, internalization and degradation of ox-LDL. It is known that ox-LDL plays an important role during atherogenesis, by inducing vascular endothelial cell activation and dysfunction, results in pro-inflammatory responses, oxidative stress, necrosis and apoptosis^5^. Endothelial cells apoptosis leads to an increased vascular permeability to cells and lipids, smooth muscle cell proliferation, increased coagulation and lipid accumulation, thus contributes to the development of atherosclerosis^6^. It is suspected that LOX-1 upregulation may halt and reverse the atherosclerotic lesions, through the binding, endocytosis, and proteolytic degradation of oxLDL^3^,^6^.

The *OLR1* gene consists of six exons and five introns that spans over 7-kb, which located on chromosome 12p13.1-p12.3. Several single nucleotide polymorphisms (SNPs) in the *OLR1* gene have been identified, including a c.501G > C transversion on exon 4, which results in an amino acidic substitution from lysine to asparagine at position 167 (p.K167N). This SNP was found to decrease binding and internalization of ox-LDL^7^ and has been associated with hypertension, myocardial infarction and carotid atherosclerosis^8^,^9^,^10^. More importantly, this SNP is statistically linked to the risk of ischemic stroke, but discrepancies still exist between different populations^11^,^12^,^13^,^14^.

PCSK9 was formerly known as neural apoptosis-regulated convertase 1 and characterized as the ninth member of the subtilisin family of kexin-like proconvertases. PCSK9 plays an essential role in the proteolytic maturation of several secretory proteins such as neuropeptides, growth factors, cytokines and pro-hormones^15^. The PCSK9 plays an important role in modulating the plasma levels of low density lipoprotein cholesterol (LDL-C) through a post-transcriptional mechanism. PCSK9 binds to low density lipoprotein receptor (LDLR) and disrupts its endocytic recycling or directs it for lysosomal degradation^16^,^17^. Therefore, PCSK9 activation can downregulate LDLR expression and inhibit the uptake of LDL-C, which in turns leading to hypercholesterolemia and ischemic stroke event^4^,^18^.

The *PCSK9* gene is located on chromosome 1p32.3 and is 22-kb in length. It comprises of 12 exons and 11 introns, which encodes for 692 amino acids. Previous studies have investigated the relationship between *PCSK9* SNPs and their changes in circulating LDL-C levels. A common SNP - 23968A > G (rs505151) in exon 12, results in an amino acid substitution from glutamate to glycine at position 670 (p.E670G), is potentially associated with the altered enzyme activity of PCSK9^19^. Moreover, this SNP has been reported to be associated with the risk of ischemic stroke, but the positive significance towards ischemic stroke event needs to be confirmed^20^,^21^,^22^.

Both OLR1 and PCSK9 are positively linked, where the inhibition of PCSK9 can suppress the development of atherosclerosis by disrupting LOX-1 expression^23^. Thus far, no meta-analysis has yet been conducted to investigate the relationship between *OLR1* rs11053646 and *PCSK9* rs505151 polymorphisms and ischemic stroke. Therefore, we undertook the current meta-analysis from all eligible case-control studies and performed a critical review on these published articles, for the purpose of providing the highest level of evidence on their risk significance.

## Results

### Studies selection and characteristics

**Figure 1:**
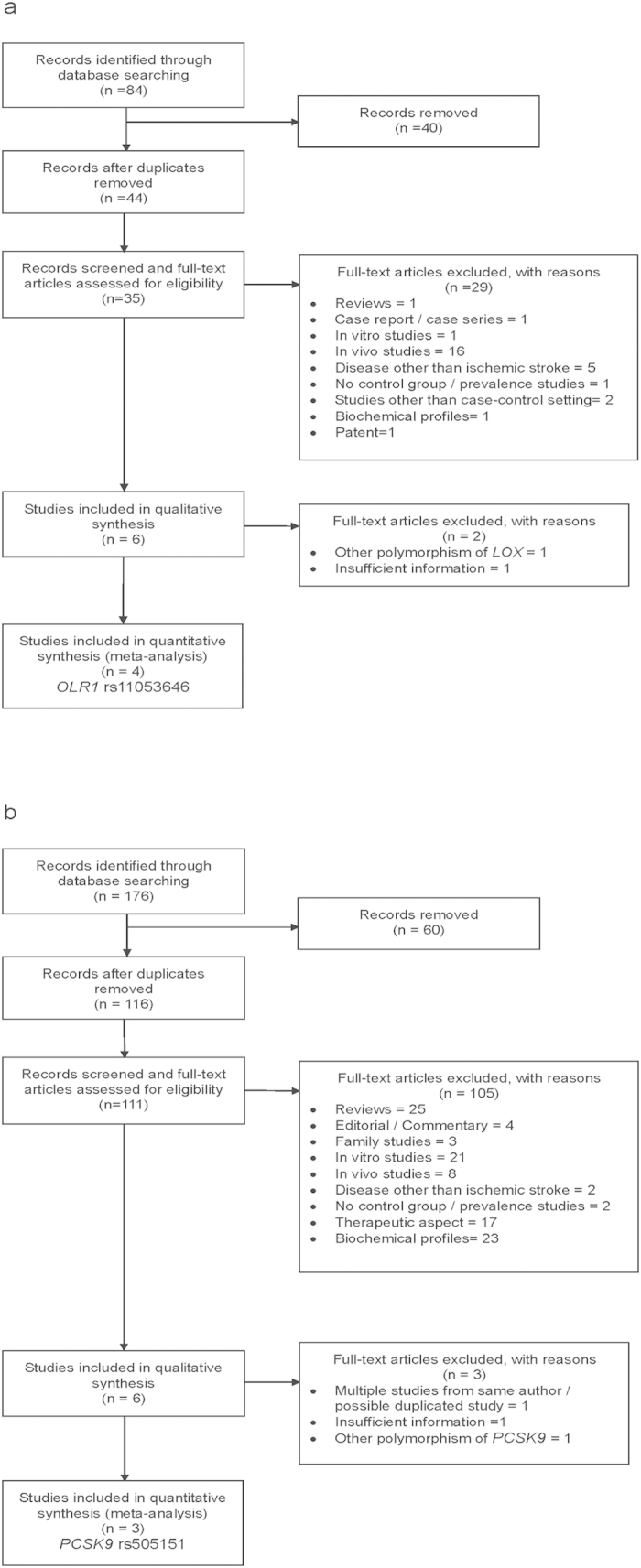
Flow chart of the study selection process for *OLR1* rs11053646 (**a**) and *PCSK9* rs505151 (**b**).

With regard to *OLR1* rs11053646 and *PCSK9* rs505151 polymorphisms, 84 and 176 studies were identified from the initial search (Fig. 1). Of these, six articles and one thesis were found to be related to the association between the studied polymorphisms and the risk of ischemic stroke. As for *OLR1* rs11053646, one study was excluded due to insufficient information while another study was reporting on polymorphism other than our interest (Fig. 1). For *PCSK9* rs505151, three studies were excluded due to (i) multiple studies from the same author (n = 1), (ii) insufficient information (n = 1), and (iii) study that reporting on polymorphism other than our interest (n = 1). No additional eligible article was found despite performing the extensive manual search on the references cited in the eligible publications and review articles. Therefore, a total of four (*OLR1* rs11053646) and three articles (*PCSK9* rs505151) encompassing 1138 and 759 cases as well as 1213 and 906 controls that met the inclusion criteria were included in the final meta-analysis model (Fig. 1). The detailed characteristics of all the selected studies were presented in Table 1. Meanwhile, the distribution of allele and genotype for each individual study were demonstrated in Table 2.

### Quantitative synthesis of data

**Figure 2:**
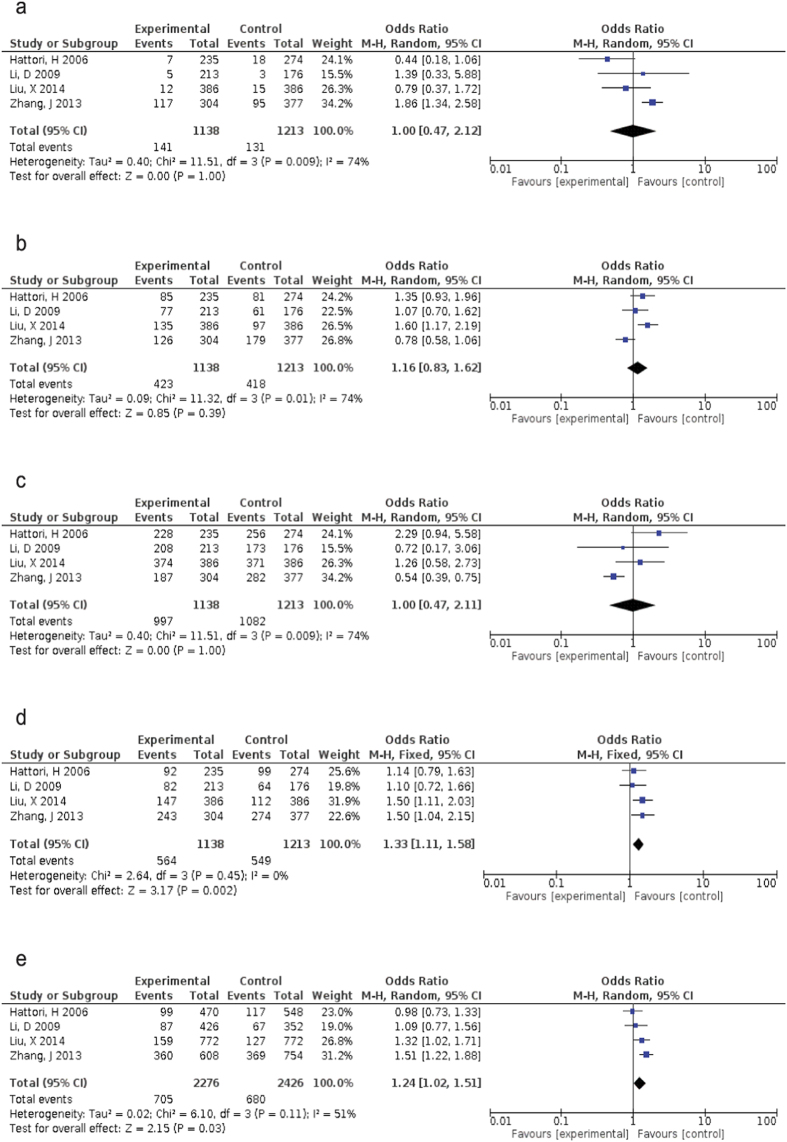
Forest plot of odds ratios for the association between *OLR1* rs11053646 and ischemic stroke risk. (**a**) under homozygous model (CC vs GG); (**b**) under heterogeneous model (GC vs GG); (**c**) under recessive model (GG + GC vs CC); (**d**) under dominant model (GC + CC vs GG); (**e**) under co-dominant model (C vs G).

**Figure 3:**
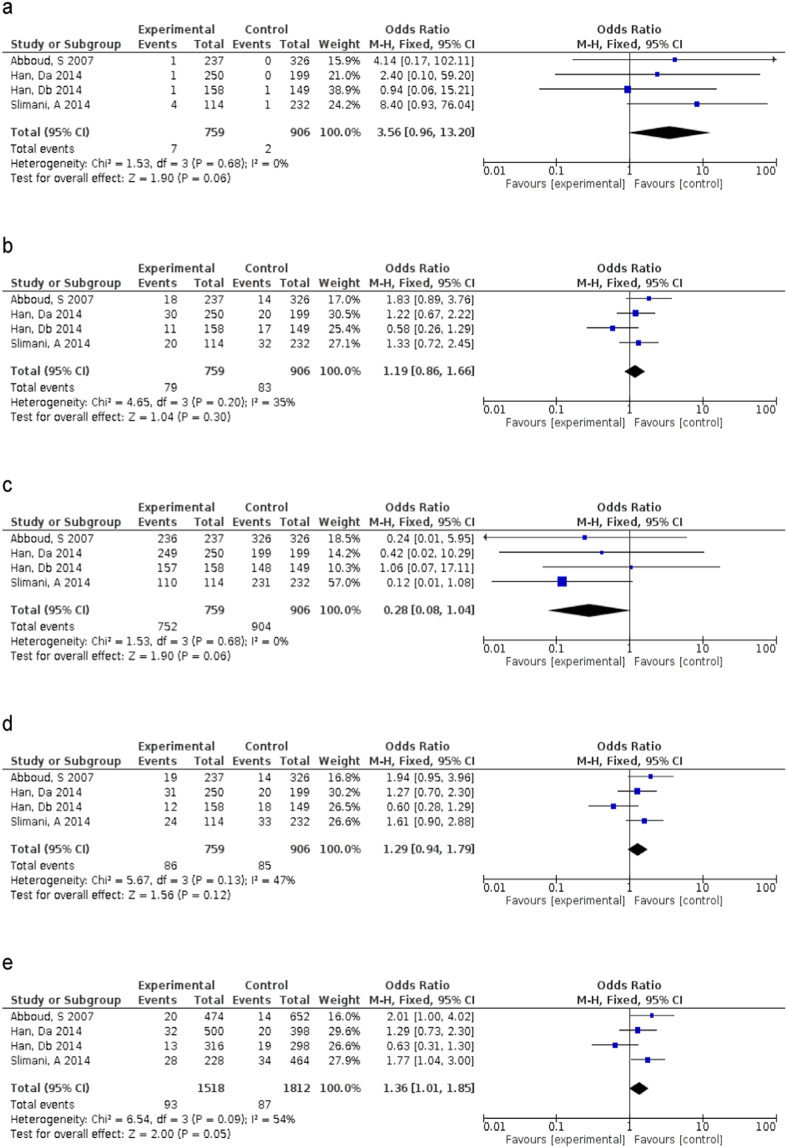
Forest plot of odds ratios for the association between *PCSK9* rs505151 and ischemic stroke risk. (**a**) under homozygous model (GG vs AA); (**b**) under heterogeneous model (AG vs AA); (**c**) under recessive model (AA + AG vs GG); (**d**) under dominant model (AG + GG vs AA); (**e**) under co-dominant model (G vs A).

Since heterogeneity has been observed in the overall comparison, random-effect models were applied for all of the forest plots (Fig. 2a–c), except for the *OLR1* rs11053646 dominant and co-dominant models (Fig. 2d–e). Ironically, the majority of genetic models for *PCSK9* rs505151 were fixed-effect models, except for its recessive genetic model (Fig. 3a–e). Nevertheless, the dominant model of *OLR1* rs11053646 was significantly increased the risks towards ischemic stroke with odd ratio (OR) 1.33 (95% CI:1.11–1.58, *p* = 0.002). Interestingly, both of the co-dominant models for *OLR1* rs11053646 and *PCSK9* rs505151 demonstrated similar odds towards ischemic stroke (OR = 1.24, 95% CI:1.02–1.51, *p* = 0.03; OR = 1.36, 95% CI:1.01–1.85, *p* < 0.05, respectively). Although it is not statistically significant, the GG genotype carriers of *PCSK9* rs505151 had a higher risk of ischemic stroke as compared to the wild-type carriers (OR = 3.56, 95% CI:0.96–13.20, *p* = 0.06).

### Heterogeneity and publication bias

**Figure 4:**
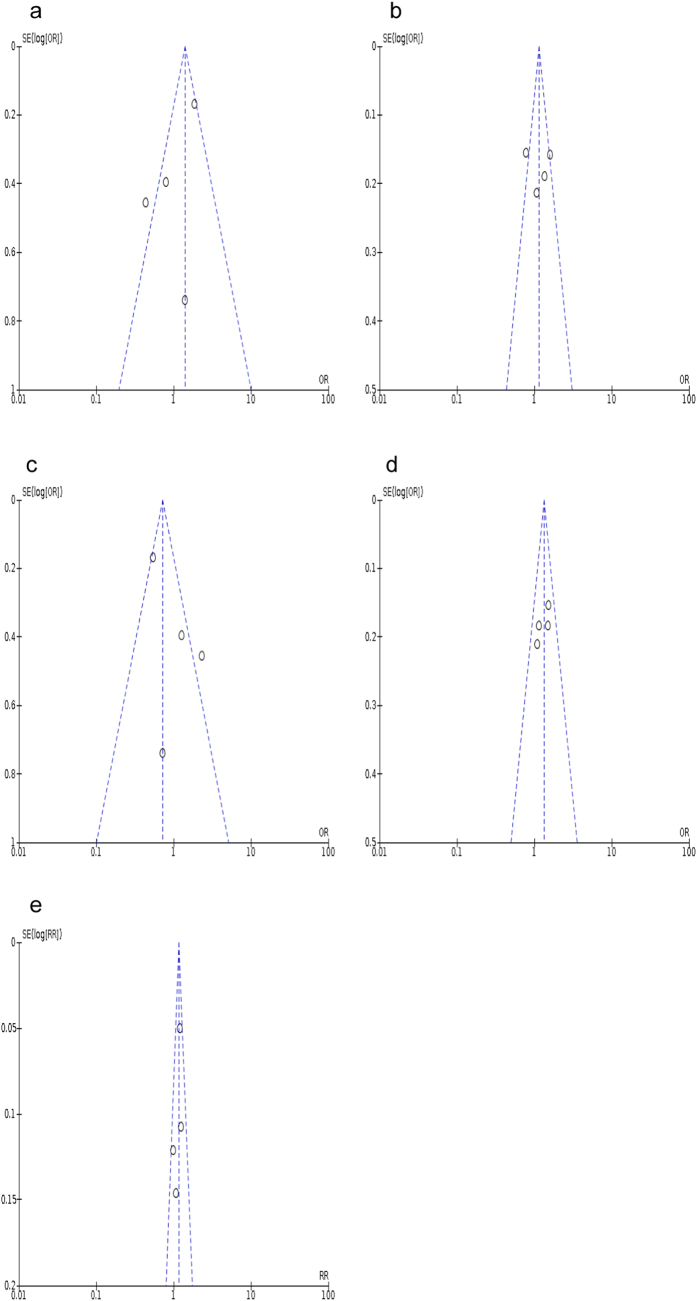
Funnel plot of publication bias for the association between *OLR1* rs11053646 and ischemic stroke risk. (**a**) under homozygous model (CC vs GG); (**b**) under heterogeneous model (GC vs GG); (**c**) under recessive model (GG + GC vs CC); (**d**) under dominant model (GC + CC vs GG); (**e**) under co-dominant model (C vs G).

**Figure 5:**
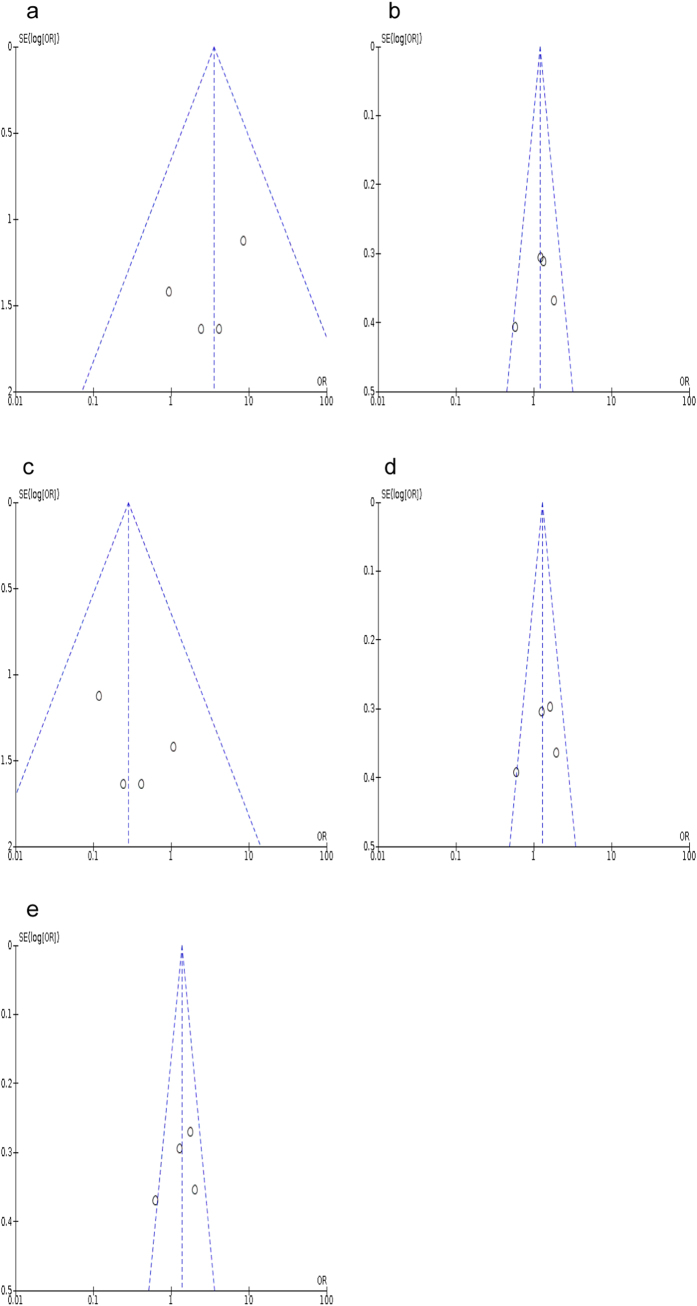
Funnel plot of publication bias for the association between *PCSK9* rs505151 and ischemic stroke risk. (**a**) under homozygous model (GG vs AA); (**b**) under heterogeneous model (AG vs AA); (**c**) under recessive model (AA + AG vs GG); (**d**) under dominant model (AG + GG vs AA); (**e**) under co-dominant model (G vs A).

The significance of inter-study heterogeneity in the overall comparison models is summarized as P_het_ < 0.10 and I^2^ > 50% (Table 3). Meanwhile, the potential publication bias was analyzed by performing both of the Begg’s and Egger’s tests. The shapes of funnel plots were relatively symmetry, except for the *OLR1* rs11053646 homozygous, heterozygous and recessive models (Fig. 4) as well as the *PCSK9* rs505151 co-dominant model (Fig. 5). However, the results from Egger’s test showed no significance of publication bias for all the tested genetic models (p > 0.05), suggesting that publication bias is not existed in this meta-analysis model. In particular, a non-significant p-value of 0.52 was observed for the co-dominant model of *PCSK9* rs505151. Likewise, no significant evidence of publication bias were identified under *OLR1* rs11053646 homozygous (*p* = 0.31) and recessive (*p* = 0.37) models, except for the heterozygous model (*p* = 0.002).

## Discussion

To the best of our knowledge, this is the first meta-analysis that comprehensively assessed the association between *OLR1* rs11053646 and *PCSK9* rs505151 polymorphisms with the risk of ischemic stroke. In this study, a total of seven eligible articles comprising 1897 stroke cases and 2119 healthy controls were included. The present meta-analysis covered all the publications indexed in the major databases such as PubMed, Scopus and Web of Science, as well as other databases from China, Hong Kong, India, Japan, Korea, Malaysia, Russia and Latin America. The positive association between the allelic variant of the studied genes and increased ischemic stroke risks represent the major findings of this meta-analysis. Present study has extended our previous knowledge on the participation of a large number of candidate genes in the development of ischemic stroke, particularly the genes involved in the coagulation, homocysteine and lipid signaling pathways^2^.

The clinical impact of *OLR1* in the pathogenesis of atherosclerosis and ischemic stroke has been investigated^3^. A higher LOX index was reported to be positively associated with ischemic stroke risk^24^. The SNP rs11053646 has been reported to be associated with the precursors of ischemic stroke such as carotid atherosclerotic plaque, intima-media thickness and left ventricular hypertrophy^25^,^26^,^27^. Functional screening and *in vitro* analysis has demonstrated that amino acids substitution of p.K167N (c.501G > C) may reduce the binding affinity of the *OLR1* receptor and reduce the LOX-1 expression^7^. In human subjects, the CC variant possesses a lower binding affinity towards ox-LDL and reduced its mRNA expression, which can lead to increased inflammation and affect the atherogenic process in the carotid artery^26^,^28^. In contrast, an 11-year follow-up study reported that plasma soluble LOX-1 levels were elevated in the CC genotype carriers as compared to GG genotype among Japanese^24^. In this meta-analysis, *OLR1* rs11053646 C allele is associated with ischemic stroke in the dominant model and/or in the recessive model. Consistent with this phenomenon, Liu and colleagues^14^ reported that the CC + GC genotype and C allele of this SNP increased the risks of ischemic stroke in Chinese population (OR = 1.51, *p* < 0.001; OR = 1.32, *p* = 0.04, respectively). Similarly, Zhang *et al.*^13^ found that C allele (OR = 1.52, *p* < 0.001) and CC genotype (OR = 2.08, *p* = 0.001) were significantly higher among Chinese patients with ischemic stroke. However, other studies have shown a lack of association between this SNP and ischemic stroke^11^,^12^. Hattori *et al.*^11^ suggested the CC + GC genotype and C allele were less likely to be associated with ischemic stroke (OR = 1.14, *p* = 0.48; OR = 0.98, *p* = 0.91, respectively). Likewise, a relatively small number of study^12^ has indicated the higher frequencies of CC genotype (OR = 1.33) and C allele (OR = 1.09) among ischemic stroke patients, but the differences with controls were not statistically significant (*p* = 0.66 and *p* = 0.63). Hence, the presence of genetic heterogeneity and small sample size could possibly explain the divergent results of these studies.

Increasing evidence has indicated the critical roles of *PCSK9* in the risk of hypercholesterolemia and ischemic stroke^4^. Recently, PCSK9 inhibitor shows promising results for the treatment of familial hypercholesterolemia and significantly reduced the LDL-C levels^29^,^30^,^31^. It is generally well accepted that genetic polymorphisms in *PCSK9* gene can contribute to the variable expression and affect the enzyme activity of *PCSK9*. Nevertheless, the association between p.E670G and LDL-C still remains controversial. Some studies^21^,^32^,^33^,^34^ have reported positive associations between G allele and increased levels of LDL-C, whereas other study^35^ has shown contrary finding. Moreover, *PCSK9* 670 GG variant is associated with higher LDL-C levels and increased intima-media thickness progression, in the presence of ApoE4 allele^34^. In contrast, several studies suggested that LDL-C levels are not mediated by this SNP^36^,^37^,^38^. With regards to the association of disease, this meta-analysis revealed that the co-dominant model of *PCSK9* rs505151 is associated with ischemic stroke, where the distribution of G allele is higher among ischemic stroke patients. Among the included studies, Abboud and colleagues^20^ first demonstrated a potential association of this SNP with ischemic stroke, especially the large-vessel atherosclerosis stroke. The odd ratios of G allele and AG + GG genotype were higher among Belgian ischemic stroke subjects (OR = 2.10, *p* = 0.047; OR = 2.01, *p* = 0.045 respectively). This observation is further supported by a Tunisian case-control study, where the incidence of G alleles (OR = 1.77, *p* = 0.032) tends to be higher towards ischemic stroke risk^21^. Interestingly, patients with AG and AA genotypes have their LDL-C levels twice as high as the normal control subjects, which indicated the association between this SNP and the risk of ischemic stroke is mediated by increased levels of LDL-C. However, the odd ratios of G allele were divergently reported among Hans (0.73) and Uygur (0.63) populations^22^.Their study suggested that there is no significant association between rs505151 and ischemic stroke, but the LDL-C levels have not been determined^22^. It is noteworthy that the true effect of this SNP could be masked by other genes or environmental factors.

A pertinent source of bias in the meta-analysis is that the source of selected study may be skewed due to the tendency of journals in selectively publishing studies with positive findings. Begg’s funnel plots demonstrated the existence of publication bias in different genetic models of *OLR1* rs11053646 and *PCSK9* rs505151. However, the non-significant P-value of Egger’s test (*p* > 0.05) indicated that the overall pooled results are unbiased, except for the *OLR1* rs11053646 heterozygous model. Therefore, the results of this model shall be interpreted with caution. The observed heterogeneity may be attributed by the different ethnicities, sample size, study design, genotyping methods, and other environmental factors. Moreover, population heterogeneity may be derived from the genetic diversity of individual studies, i.e. genetic diversity may still exist even though the studied subjects are derived from the same population, ethnicity, country and district.

However, there are several limitations in the current meta-analysis. For instance, we did not perform stratification analysis according to ischemic stroke subtypes since TOAST classification was not been reported in any of the eligible studies. In addition, the subgroup analysis has not been carried out in this meta-analysis. The population data of *PCSK9* rs505151 are limited for the subgroup analysis, since only a single Asian population was presented in this meta-analysis. Despite these limitations, our meta-analysis covered the most available association case-control studies, which involving both the hospital- and population-based. Furthermore, the present study has provided a better understanding on the association between the studied SNPs and the risk of ischemic stroke for the first time.

In conclusion, the current meta-analysis suggested that the variant alleles of *OLR1* rs11053646 and *PCSK9* rs505151 may confer an increased risk of ischemic stroke. Therefore, these SNPs may be used as genetic biomarkers in relation to the burden of ischemic stroke, and serve as potential targets for diagnostic and therapeutic implications, and could thus have potential benefit for translational research in ischemic stroke. Further investigations with larger number of samples from more countries are needed, in order to facilitate the translation of genetic biomarkers into clinical practice.

## Methods

### Search strategy

This meta-analysis followed the Cochrane Collaboration definition and PRISMA 2009 guidelines for meta-analysis and systematic review. We performed a comprehensive literature search throughout PubMed, Scopus, Web of Science, Google scholar, WHO Global Health Library, VHL, Jstage, KoreaMed, Korean Science Citation Index, POPLINE, New York Academy of Medicine Grey Literature Report, Indian Citation Index, System for Information on Grey Literature in Europe, IMSEAR, MJM, Mycite, WPRIM and CNKI to retrieve the genetic association studies of ischemic stroke. The medical subject heading and keywords terms “lectin-like oxidized LDL receptor-1”, “*LOX-1*”, “*OLR1*”, “proprotein convertase subtilisin/kexin type 9”, “PCSK9”, “neural apoptosis-regulated convertase 1”, “NARC1”, “ischemic stroke”, “cerebrovascular disease”, “cerebrovascular accident”, “brain infarction”, “brain ischemia”, “cerebral ischemia”, polymorphism”, “variant”, “gene mutation”, “single nucleotide polymorphism (SNP)”, “gene variation” and the related Chinese characters were used as the criteria for searching. There was no limitation in language, where articles written in English, Japanese, Korean, Spanish, Russian or Chinese were retrieved. In addition, the time period for literature searching was from the first available article until July 2015.

### Study selection and data abstraction

The inclusion criteria for the gene association studies in the final meta-analysis were as follows: (i) case-control and/or cohort studies; (ii) contained SNP genotype data; and (iii) adequate data for the calculation of odds ratios (ORs) and 95% confidence intervals (CIs).

Data abstraction was performed independently by two authors (A.A. and L.K.W.). This meta-analysis was conducted when the data of three unduplicated studies are available. The following information from each study was summarized: (i) first author; (ii) publication year; (iii) province of study population; (iv) ethnicity; (v) number of cases and controls; (vi) mean age and sex ratio; and (vii) genotyping method.

### Statistical analysis

The genotypic distributions for studied polymorphisms were compared against the controls for any possible deviations from the Hardy-Weinberg equilibrium. Crude OR and 95% confident interval (CIs) were calculated to test the strength of associations between studied polymorphisms and ischemic stroke. The significance of the pooled ORs was determined by Z test for polymorphisms under different genetic models (homozygous, heterozygous, recessive, dominant and co-dominant) for *OLR1* rs11053646 and *PCSK9* rs505151. The heterogeneity for all the included studies was evaluated using Cochran’s Q test and I^2^ statistics. The random-effects model was chosen when significant heterogeneous exist (P_heterogeneity_ < 0.05, I^2^ > 50%); otherwise, fixed-effects model would be adopted. Potential publication bias was tested with Begg’s funnel plot and Egger’s regression test. The statistical tests were performed using the Review manager version 5.3 and Metafor package in R version 3.2.1^39^. All statistics were two-sided and *p* < 0.05 was considered statistically significant.

## Additional Information

**How to cite this article**: Au, A. *et al.* The Influence of *OLR1* and *PCSK9* Gene Polymorphisms on Ischemic Stroke: Evidence from a Meta-Analysis. *Sci. Rep.* **5**, 18224; doi: 10.1038/srep18224 (2015).

## Figures and Tables

**Table 1:** Main characteristic of the studies included in the current meta-analysis.

Author	Year	Country	Ethnicity	Total no. of		Mean age		Sex (M/F)		Control Origin	Genotyping method
				Case	Control	Case	Control	Case	Control		
Abboud, S	2007	Belgium	Caucasians	237	326	53.5	70.3	2.0	2.0	Population-based	TaqMan SNP genotyping assay
Han, D^a^	2014	China	Asians	250	199	63.6 ± 11.3	62.4 ± 11.7	2.5	1.1	Hospital-based	Single-base terminalextension
Han, D^b^	2014	China	Caucasians	158	149	59.4 ± 12.0	61.2 ± 11.5	1.6	1.2	Hospital-based	Single-base terminalextension
Hattori, H	2006	Japan	Asians	235	274	58.3 ± 7.8	59.1 ± 3.4	3.5	2.5	Hospital-based	Single-nucleotide primer extension
Li, D	2009	China	Asians	213	176	60.0 ± 13.8	59.0 ± 11.4	1.2	1.2	Hospital-based	Restriction fragment length polymorphism
Liu, X	2014	China	Asians	386	386	62.1 ± 9.9	61.9 ± 9.8	2.2	2.2	Hospital-based	Ligation detection reaction
Slimani, A	2014	Tunisia	Caucasians	114	232	66 (54.5–76.5)	49.0 (45.0–55.0)	1.4	2.9	Hospital-based	Restriction fragment length polymorphism
Zhang, J	2013	China	Asians	304	377	61.2 ± 7.1	61.1 ± 6.9	1.5	1.6	Population-based	Restriction fragment length polymorphism

^a^ and ^b^ are from the same study, Han D *et al.* (2014).

**Table 2:** The distribution of alleles and genotypes of *OLR1* rs11053646 and *PCSK9* rs505151 polymorphisms in the current meta-analysis.

Studied Polymorphisms	Author	Year	Sample size		Case					Control					HWE
			Case	Control	W	H	V	D	M	W	H	V	D	M	p value
OLR1 rs11053646	Hattori, H	2006	235	274	143	85	7	371	99	175	81	18	431	117	0.05
	Li, D	2009	213	176	131	77	5	339	87	112	61	3	285	67	0.10
	Liu, X	2014	386	386	239	135	12	613	159	274	97	15	645	127	0.09
Zhang, J	2013	304	377	61	126	117	248	360	103	179	95	385	369	0.33	
*PCSK9* rs505151	Abboud, S	2007	237	326	218	18	1	454	20	312	14	0	638	14	0.69
	Han, D^a^	2014	250	199	219	30	1	468	32	179	20	0	378	20	0.46
	Han, D^b^	2014	158	149	146	11	1	303	13	131	17	1	279	19	0.59
Slimani, A	2014	114	232	90	20	4	200	28	199	32	1	430	34	0.81	

HWE: Hardy-Weinberg Equilibrium; W: wild type; H: heterozygous; V: variant; D: dominant allele frequency; M: minor allele frequency.

**Table 3:** Meta-analysis of the association between *OLR1* rs11053646 and *PCSK9* rs505151 with ischemic stroke.

Variables	No. of study	Sample size (cases/controls)	Homozygous				Heterozygous				Recessive				Dominant				Co-dominant			
			OR (95% CI)	P	P het	I^2^ (%)	OR (95% CI)	P	P het	I^2^ (%)	OR (95% CI)	P	P het	I^2^ (%)	OR (95% CI)	P	P het	I^2^ (%)	OR (95% CI)	P	P het	I^2^ (%)
*OLR1* rs11053646 G > C	4	1138/1213	CC vs. GG				GC vs. GG				CC vs. (GG + GC)				(GC + CC) vs. GG				C vs. G			
			1.00 (0.47–2.12)	1.00	0.009	74	1.16 (0.83–1.62)	0.39	0.01	74	1.00 (0.47–2.11)	1.00	0.009	74	1.33 (1.11–1.58)	0.002	0.45	0	1.24 (1.02–1.51)	0.003	0.11	51
*PCSK9* rs505151 A > G	3	759/906	GG vs. AA				AG vs. AA				GG vs. (AA + AG)				(AG + GG) vs. AA				G vs. A			
			3.56 (0.96–13.20)	0.06	0.68	0	1.19 (0.86–1.66)	0.30	0.20	35	1.61 (0.04–67.16)	0.06	0.68	0	1.29 (0.94–1.79)	0.12	0.13	47	1.36 (1.01–1.85)	0.05	0.09	54

P, p-value for Z test; P ^het^, p-value for Cochrane’s Q test.
